# Exploring the natural history of intrinsic capacity impairments: longitudinal patterns in the 10/66 study

**DOI:** 10.1093/ageing/afad137

**Published:** 2023-07-29

**Authors:** Emmanuel Gonzalez-Bautista, Jorge Jesus Llibre-Guerra, Ana L Sosa, Isaac Acosta, Sandrine Andrieu, Daisy Acosta, Juan de Jesús Llibre-Rodríguez, Matthew Prina

**Affiliations:** Maintain Aging Research Team, CERPOP, Université de Toulouse, Inserm, Université Paul Sabatier, Toulouse, France; Institute on Aging, Toulouse University Hospital (CHU), Gerontopole, Toulouse, France; Department of Health Service & Population Research, King’s College London, Institute of Psychiatry, Psychology &Neuroscience, London, UK; Department of Neurology, Washington University School of Medicine, St. Louis, MO, USA; National Institute of Neurology and Neurosurgery of Mexico, National Autonomous University of Mexico, Mexico City, Mexico; Internal Medicine Department, Geriatric Section, Universidad Nacional Pedro Henriquez Ureña, Santo Domingo, Dominican Republic; Maintain Aging Research Team, CERPOP, Université de Toulouse, Inserm, Université Paul Sabatier, Toulouse, France; Internal Medicine Department, Geriatric Section, Universidad Nacional Pedro Henriquez Ureña, Santo Domingo, Dominican Republic; Facultad de Medicina Finlay-Albarran, Medical University of Havana, Havana, Cuba; Department of Health Service & Population Research, King’s College London, Institute of Psychiatry, Psychology &Neuroscience, London, UK; Faculty of Medical Sciences, Population Health Sciences Institute, Newcastle University, Newcastle Upon Tyne, UK

**Keywords:** intrinsic capacity, natural history of functional decline, latent transitions, longitudinal analysis, older people

## Abstract

**Background:**

intrinsic capacity (IC) is a construct encompassing people’s physical and mental abilities. There is an implicit link amongst IC domains: cognition, locomotion, nutrition, sensory and psychological. However, little is known about the integration of the domains.

**Objectives:**

to investigate patterns in the presentation and evolution of IC domain impairments in low-and-middle-income countries and if such patterns were associated with adverse outcomes.

**Methods:**

secondary analyses of the first two waves of the 10/66 study (population-based surveys conducted in eight urban and four rural catchment areas in Cuba, Dominican Republic, Puerto Rico, Venezuela, Peru, Mexico and China). We applied latent transition analysis on IC to find latent statuses (latent *clusters*) of IC domain impairments. We evaluated the longitudinal association of the latent statuses with the risk of frailty, disability and mortality, and tested concurrent and predictive validity.

**Results:**

amongst 14,923 participants included, the four latent statuses were: high IC (43%), low deterioration with impaired locomotion (17%), high deterioration without cognitive impairment (22%), and high deterioration with cognitive impairment (18%). A total of 61% of the participants worsened over time, 35% were stable, and 3% improved to a healthier status.

Participants with deteriorated IC had a significantly higher risk of frailty, disability and dementia than people with high IC. There was strong concurrent and predictive validity. (Mortality Hazard Ratio = 4.60, 95%CI 4.16; 5.09; Harrel’s C = 0.73 (95%CI 0.72;0.74)).

**Conclusions:**

half of the study population had high IC at baseline, and most participants followed a worsening trend. Four qualitatively different IC statuses or statuses were characterised by low and high levels of deterioration associated with their risk of disability and frailty. Locomotion and cognition impairments showed other trends than psychological and nutrition domains across the latent statuses.

## Key Points

- Literature on the relationship between the intrinsic capacity domains is scarce.
- Our study identified and validated four latent statuses or clusters of intrinsic capacity impairments.
- Both the deterioration level and the type of domain affected (notably cognition and locomotion) are linked to clinical outcomes.
- About 3% of the study population transitioned to a higher intrinsic capacity status.

## Background

Intrinsic capacity (IC) is the aggregate of physical and mental capacities people can draw upon as they age [1]. Understanding IC is crucial for healthy longevity because it informs about the preservation of central physiological systems that operate to allow older adults to be functional. The concept and measurement of IC are under construction as there is no consensus on a definitive IC operationalisation.

Studies have found that IC encompasses the locomotive, cognitive, psychological, nutritional and sensory (vision and hearing) domains [2–4]. The authors used a reflective structural IC model, which implies that one could remove one of the domains and still be able to measure IC and that the correlations amongst the domains should be high [5]. However, those implications have not been sufficiently discussed in the ageing field.

The construct of IC is relevant from the research and the healthcare points of view. Assessing IC in care settings is helpful as an indicator of the core physiological elements that allow older adults to be and do what is meaningful for them [6, 7]. Monitoring IC in individuals and populations enables the detection of early functional declines for timely interventions [3, 8, 9], as has been demonstrated in the INSPIRE-Icope care cohort in the south of France [10].

If IC permeates the health systems, it might help to switch the disease-centred paradigm into a person-centred one. Re-organising health services using the IC domains and its clinical pathways might improve the healthcare provision for older people [6]. For instance, the World Health Organisation’s Integrated Care for Older People (ICOPE) is a strategy to foster joined-up care for older adults [11]. Publications from China, [12] France, [7] Hong Kong [13] and Taiwan [14] have used ICOPE to assess IC in older adults. However, evidence from low-and-middle-income countries (LMIC) is still limited.

Older adults are likely to be impaired in more than one IC domain [7, 15]. For instance, the percentage of older adults with at least one IC domain impairment ranged between 43% and 94%, with a mean of 74% in eight previous publications from around the world [7, 12, 13, 15–19].

It would be helpful to know the presentation pattern of those impairments. Multidomain interventions could be designed and planned to target the most common patterns. So far, studies have reported clusters of IC domain impairments only cross-sectionally and circumscribed to one country [21, 22]. Examination of data from the global north and the global south could expand our knowledge of the natural history of IC impairments in older adults.

Therefore, this study aims to investigate patterns in the presentation and evolution of IC domain impairments in LMIC using data from the 10/66 dementia research group (10/66 Dementia Research Group (DRG)) study and if such patterns are associated with adverse outcomes and assert their concurrent and predictive validity.

## Methods

### Participants

The 10/66 DRG population-based studies of ageing and dementia included people aged 65 years and over living in selected geographic areas of Peru (urban Lima and rural Canete), Mexico (urban Mexico City and rural Morelos state), China (urban Xicheng and rural Daxing) and India (urban Chennai and rural Vellore), and urban sites in Cuba (Havana/Matanzas), Dominican Republic (Santo Domingo), Puerto Rico (Bayamon) and Venezuela (Caracas).

Baseline surveys were carried out between 2003 and 2007, and the follow-up between 2008 and 2010 in most centres. A detailed description of the 10/66 cohorts has been published elsewhere [23, 25]. We did not include India data because follow-up was unavailable. The sample of the rest of the countries was included in the final analyses.

The baseline cohort was defined by systematically door-knocking all households in each selected geography (catchment area). Eligibility criteria: residents aged 65 and over living in the catchment area. No direct exclusion criteria were applied, but the design excluded high-income urban districts.

Data was gathered via household interviews, including medical history, healthcare utilisation and lifestyle-related factors; a cognitive assessment; a physical examination; and an informant interview. The King’s College London and the local research ethics committee approved the study.

Informed consent was collected on paper, and literate participants signed their approval. The information sheet was read to illiterate participants by a literate, independent witness who provided attestation. For participants without the capacity to consent, agreement for their participation was obtained from next of kin. The ethics committees approved these procedures.

## Measures

### Intrinsic capacity impairments

We followed the same approach as the previously published paper by Prince and coauthors [17].

Locomotion: walking speed <0.8 m/s. Walking speed was assessed by a timed walking test (5 m at their usual pace, turn and return to the starting point).

Nutrition: self-report of weight loss of ≥4.5 kg in the last 3 months, or if their mid-upper arm circumference was measured to be <22 cm [26].

Vision: Self-report of ‘eyesight problems’ that affected their activities or if they were identified as being functionally blind by the interviewer.

Hearing: Self-report of ‘hearing problems or deafness’ interfered with their activities or if the interviewer identified them as deaf.

Cognition: scoring <29.5 in the Community Screening Instrument for Dementia (CSI-D) COGSCORE, which tests multiple domains of cognitive function and has been found to have robust cross-cultural measurement properties in the 10/66 DRG study sites. Scores below that threshold identify cases of cognitive impairment [27].

Psychological: Endorsing >3 of the 12 depression symptoms in the EURO-D depression scale. This cut-point identified individuals with subsyndromal depression or depressive symptoms that did not meet the criteria for a depressive episode, such as ICD–10 o DSM-IV [28, 29].

### Outcomes

Frailty: Fried’s phenotype adapted to data availability [30]. From the original five frailty components (exhaustion, weight loss, weak grip strength, slow walking speed and low energy expenditure), grip strength was not available in 10/66. We considered participants frail if they exhibited two or more of the four available components, as used in previous reports [31–33].

Dementia: cross-culturally validated 10/66 DRG dementia diagnosis algorithm [27]. This algorithm was derived from the community screening instrument for dementia (CSI ‘D’) [34] and the geriatric mental state (GMS/AGECAT) [35].

Disability: World Health Organisation Disability Assessment Schedule version 2.0 (WHODAS 2.0). The 12-item WHODAS 2.0 has good clinimetric properties, and a recent review has concluded that it is suitable for assessing disability in various settings and populations [36–38]. The questionnaire has been included as Supplementary Material S2.

Self-rated health was assessed with the question, ‘How do you rate your overall health in the past 30 days?’ [39].

Mortality: Vital status at follow-up was verified at the participant’s households using a verbal autopsy, including the date and cause, if available.

The outcomes were collected during the household visit by trained interviewers.

### Covariates

Education: Self-reported highest level of formal education achieved.

Comorbidities: Count how many from the following: dementia as described above, [27] depression (algorithm from the clinical interview based on ICD-10), diabetes (self-reported), hypertension (ascertained blood pressure, systolic blood pressure ≥140 mm and/or diastolic blood pressure ≥ 90 mm Hg and self-report of previous diagnosis and treatment), and self-reported stroke.

### Statistical analyses

First, we obtained the baseline prevalence of IC domain impairments from the participating countries. Second, we applied the latent transition model to the IC impairments. The latent transition model finds unobserved ‘profiles’ of people with a similar combination of domain impairments called ‘statuses’. The latent statuses consider the quantity and quality of the domains affected. Participants with a given latent status have the same average probability of being impaired in the IC domains. The model allows these profiles to be consistent at both time points and estimates the transition from baseline latent profiles to those at follow-up.

The latent transitions model generated four categories (statuses), our main independent variables. We opted for the model with four latent statuses based on the physio-clinical correlation and the model diagnostics (Figure 2 and Further methodological details in Annex S3) [24]. Then, we evaluated the predictive ability of the four latent statuses using incident outcomes with logistic regressions for frailty, dementia and disability. To enhance interpretability, we estimated the adjusted probability of the outcomes given the latent status and expressed it as a percentage. For mortality, we fitted a Cox model, obtained the Kaplan–Meier survival curve, verified the proportional hazards assumption using time-varying covariates and log–log plot methods, and estimated the Harrel’s C index. We tested the concurrent validity of the latent statuses with self-rated health (coded from 0 ‘very good’ to 4 ‘very bad’).

Analyses were performed using STATA 17® [40] and SAS 9.3 (SAS Institute, Cary, NC).

## Results

The baseline IC impairments and further description of the study sample are provided in Table 1. We included 14,923 participants with a mean age of 74.5 (SD 7.1), and 63.2% were female. The mean length of follow-up was 4.2 years (inter-quartile range (IQR) 3.0–4.9 years), with the vital status of 88.6% of the participants verified and mean time to mortality 2.6 years (IQR 1.5–3.7 years).

**Table 1:** Baseline population characteristics

	Overall *n* = 14,923		High deterioration with cognitive impairment *n* = 2,827 (18.9%)		High deterioration without cognitive impairment *n* = 2,904 (19.5%)		Low deterioration mainly locomotion impairment *n* = 2,126 (14.3%)		High IC *n* = 7,066 (47.4%)	
Age, mean (SD)	74.5	(7.1)	81.4	(7.3)	74.3	(6.2)	77	(6.3)	71.1	(4.9)
Female n (%)	9,436	(63.2)	1999	(70.7)	2,191	(75.4)	1,392	(65.5)	3,854	(54.5)
*Education n* (%)										
None	2,180	(14.6)	866	(30.6)	222	(7.6)	820	(38.6)	272	(3.8)
Some	3,866	(25.9)	1,113	(39.4)	923	(31.8)	715	(33.6)	1,115	(15.8)
Complete primary	4,366	(29.3)	577	(20.4)	993	(34.2)	473	(22.2)	2,323	(32.9)
Complete secondary	2,863	(19.2)	199	(7.0)	523	(18.0)	102	(4.8)	2039	(28.9)
High-school or above	1,645	(11.0)	70	(2.5)	243	(8.4)	16	(0.8)	1,316	(18.6)
*Comorbidities n* (%)										
0	10,521	(70.5)	1,042	(36.9)	1,483	(51.1)	1826	(85.9)	6,170	(87.3)
1	3,266	(21.9)	1,129	(39.9)	1,084	(37.3)	261	(12.3)	792	(11.2)
2+	1,136	(7.6)	656	(23.2)	337	(11.6)	39	(1.8)	104	(1.5)
*IC impairments* n (%)										
Locomotion	4,348	(32.8)	1,126	(51.6)	1,066	(41.3)	1,247	(62.8)	909	(14.0)
Nutrition	1816	(12.8)	608	(23.0)	770	(27.8)	38	(1.8)	400	(6.0)
Vision	4,529	(30.4)	1,305	(46.3)	1822	(62.7)	267	(12.6)	1,135	(16.1)
Hearing	2,369	(15.9)	847	(30.0)	966	(33.3)	203	(9.6)	353	(5.0)
Cognition	3,341	(22.4)	2,619	(92.6)	163	(5.6)	302	(14.2)	257	(3.6)
Psychological	3,466	(23.7)	1,057	(41.1)	1873	(64.7)	0	(0.0)	536	(7.6)
Frailty^a^ *n* (%)	2,530	(17.0)	970	(34.3)	963	(33.2)	260	(12.2)	337	(4.8)
Disability^a^ *n* (%)	4,360	(29.2)	1,673	(59.2)	1,239	(42.7)	491	(23.1)	957	(13.5)
Mortality *n* (%)	2,415	(18.4)	922	(37.5)	402	(15.7)	436	(22.9)	655	(10.6)

^a^At baseline.

The IC impairment with higher and lower frequency at baseline was locomotion (29.6%) and hearing (15.2%), and at follow-up, locomotion (53.9%) and nutrition (11.8%), respectively.

### Longitudinal latent statuses

**Figure 1:**
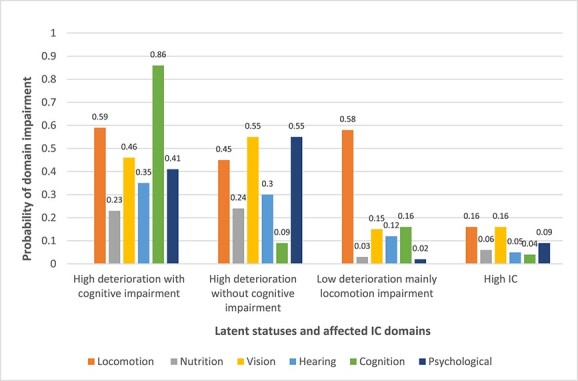
Four latent statuses of IC impairments and their domain impairments. *The four latent statuses result from the latent transitions modelling and cluster participants with similar probabilities of domain impairments. We have labelled each latent status according to the quantity and type of the IC domains affected, with high deterioration meaning at least three domains affected.*

Given the quantity and quality of the domains affected resulting from the latent status model, we assigned a name for each profile or status. (e.g. ‘high deterioration’ was used for latent profiles with three or more domains affected). The four latent statuses resulting from the latent transition modelling were: high IC (43% at baseline), low deterioration with impaired locomotion (17%), high deterioration without cognitive impairment (22%) and high deterioration with cognitive impairment (18%). The probability of exhibiting each IC domain impairment conditional on belonging to each of those statuses is shown in Figure 1. These statuses result from the model with the data at both time points and adjusting for age, sex and education level.

**Figure 2:**
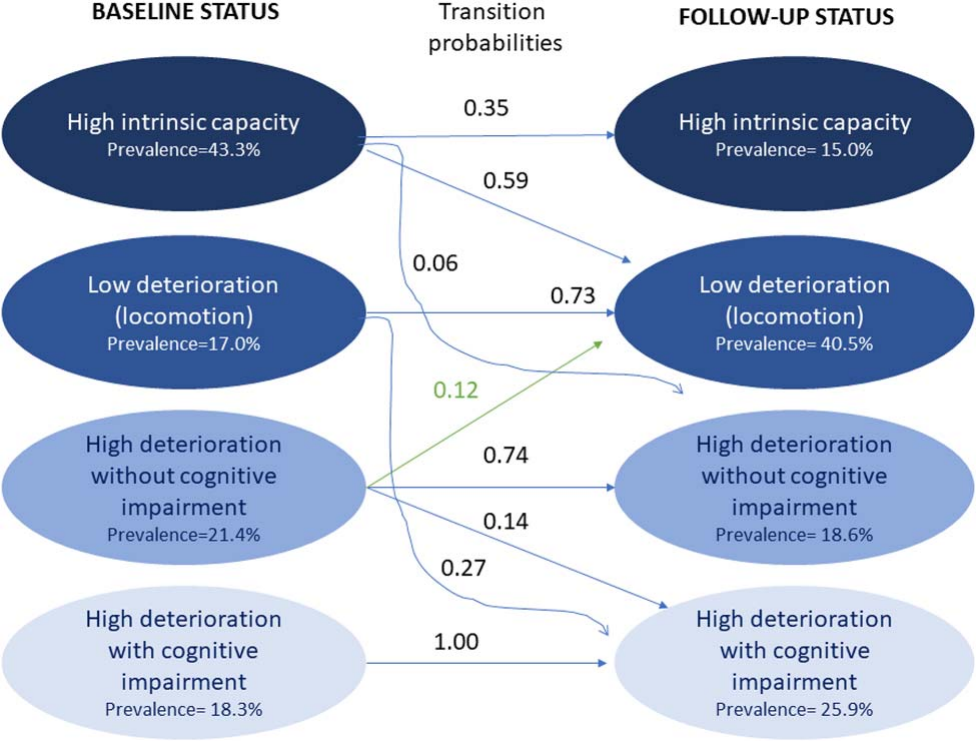
Prevalence and transition probabilities amongst the latent statuses at baseline and follow-up. The blue arrows indicate a transition to a stable or worsening latent status. Curved arrows mark transitions with an accelerated decline in IC. The green arrow signals a transition to a latent status with higher IC.

### Transitions between latent statuses

The probability of transitioning to the statuses conditional on belonging to each baseline status is shown in Table 2. The chance of transitioning to another status at follow-up was null for participants in the high deterioration with cognitive impairment status (Figure 2). Most of the low deterioration with locomotion participants remained the same at follow-up, except for 27% who abruptly changed to the high deterioration with cognitive impairment. Of those with high deterioration without cognition status, 12% improved (transited to the low deterioration status). The participants in the high IC status transitioned mainly to the low deterioration status (60%), and 6% showed a severe decline by transitioning to high deterioration without cognitive impairment. There was no reversion to the high IC status.

**Table 2:** Estimation of the risk of incident frailty, dementia, disability and mortality given the baseline latent status

	Incident frailty		Incident dementia		Incident disability		Mortality			
	*n* = 8,842		*n* = 9,215		*n* = 7,425		*n* = 12,258			
	aOR^*^	95%CI	aOR^*^	95%CI	aOR^*^	95%CI	HR	95%CI	aHR^*^	95%CI
High deterioration with cognitive impairment	3.49	(2.80;4.35)	16.94	(12.81;22.40)	4.61	(3.69;5.74)	4.6	(4.16;5.09)	1.89	(1.63;2.19)
High deterioration without cognitive impairment	3.37	(2.86;3.97)	2.82	(2.21;3.60)	2.87	(2.45;3.36)	1.61	(1.42;1.83)	1.18	(1.03;1.36)
Low deterioration mainly locomotion impairment	2.02	(1.65;2.48)	6.1	(4.69;7.94)	2.07	(1.71;2.49)	2.1	(1.86;2.37)	1.44	(1.25;1.66)
High IC	Ref.		Ref.		Ref.		Ref.		Ref.	

^*^aOR= adjusted odds ratio; HR= hazards ratio; aHR = adjusted hazards ratio.

### Concurrent validity

The IC latent statuses were significantly associated with self-rated health. Derived from the generalised ordered logistic model, the probability of reporting being in ‘very good health’ was the highest for the high IC status (Pr = 0.16, 95%CI 0.15; 0.17) and the lowest for the ‘high deterioration with cognitive impairment’ (Pr = 0.07, IC95% 0.06; 0.08). Inversely, the probability of self-rating health as ‘very bad’ was the lowest for the high IC status (Pr = 0.00, 95%CI 0.00;0.00) and the highest for the high impairment with cognitive impairment latent status (Pr = 0.05, 95%CI 0.04; 0.06). The rest of the marginal probabilities are in Supplementary Material S4.

### Predictive validity of the latent statuses of incident outcomes (frailty, disability and dementia) and mortality

Participants in the high deterioration statuses had three times the risk of frailty than people in the high IC status (high deterioration without cognitive impairment OR = 3.22 95%CI 2.75; 3.77 and high deterioration with cognitive impairment OR = 3.14 95%CI 2.58; 3.83). The low deterioration status had 82% higher chances of developing frailty at follow-up than the high IC status (OR = 1.82 95%CI 1.53;2.19) (Table 2).

The high deterioration with cognitive impairment group had the highest risk of incident dementia and incident disability (OR dementia = 17.6 95%CI 13.3; 23.15; OR disability = 4.03 95%CI 3.31; 4.90, the reference was the high IC group) (Table 2).

The adjusted probability of incident disability for the participants in each status was as follows: high deterioration with cognitive impairment status 45.2% (95%CI 41.1; 49.2), high deterioration without cognitive impairment 36.1% (95%CI 33.3; 38.8), low deterioration mainly locomotion 27.8% (95%CI 25.2; 30.3) and high IC 17.7% (95%CI 16.4; 18.9).

Mortality was nearly five times higher amongst those with high deterioration with cognitive impairment latent status than those with high IC latent status (HR = 4.60, 95%CI 4.16; 5.09), and about twofold when adjusted for age, sex, education and comorbidities (aHR = 1.89, 95%CI 1.63; 2.19). Mortality was significantly higher for all the latent statuses than for the high IC status, with a Harrel’s C index of 0.73 (95%CI 0.72;0.74) (Table 2 and Figure S1).

## Discussion

Our study is the first to explore how IC impairments change over time in a multi-country cohort and LMICs. By applying the latent class model to the IC impairments, we aim to integrate two aspects of a construct: the quantity and type of the domains affected, which resulted in the latent ‘statuses’ or ‘profiles’ of IC impairments. We found four types of older adults (four statuses), namely high IC (43% at baseline), low deterioration with impaired locomotion (17%), high deterioration without cognitive impairment (22%) and high deterioration with cognitive impairment (18%). Over half of the study sample remained in the same status at baseline and follow-up (61%). Around one-fourth of participants transitioned from the high IC to the low deterioration status, and only 3% of the participants improved their status. Interestingly, the probability of improvement was observed in the status of high deterioration (Figure 2). Participants in the latent statuses of low and high levels of deterioration had a significantly higher risk of frailty, disability and dementia than their high IC counterparts.

Our study contributes new knowledge about the natural history of IC impairments in older adults. For instance, we observed that the nutritional and psychologic domains tended to ‘move in block’. In other words, the latent statuses with higher posterior probabilities of nutritional impairment also had higher probabilities for psychological impairment. It is unclear if the overlap of these domains was driven only by nutrition measurement or if it could have a physiological background. The ICOPE Step 1 tool measured the nutritional domain with items related to appetite and weight loss [6]. Appetite loss has been previously recognised also as a depressive symptom [41, 42]. For instance, appetite loss is an item of the Centre for Epidemiologic Studies Depression Scale [43]. There are neurotransmitters involved in appetite regulation and depression (e.g. serotonin) [44, 45]. As in this example, the IC domains might share biological substrates at the cellular or molecular levels. For instance, recent work has discussed the link between IC impairments and inflammation [46]. This question deserves further research but is out of the scope of our study.

The cognitive and locomotion domains did not move in block. The cognitive and locomotion domains seem to be the ‘gatekeepers’ for transitions. For example, locomotion impairment was frequent in the transition from high IC to low deterioration, suggesting that locomotion was the beginning of the declining route for high IC adults. This observation highlights the usefulness of mobility impairment as an early clinical marker of the disabling cascade. Mobility is one of the core elements in the disabling cascade [47].

The absence of cognitive impairment in participants with high deterioration may allow them to reverse (12% of the high deterioration without cognition reversed to the low deterioration status). Reversibility was not seen for people in the high deterioration with cognitive impairment group. When clustered with the other domains, cognitive impairment seemed to mark a ‘no-return point’, almost cancelling the chances of reverting to a better status. We suggest two mechanisms: (i) transition to mortality: because of low reserves and intrinsic capacities; (ii) systemic disease and inflammation: dementia has a known extra-cerebral impact which might decrease the functional reserve [48, 49]; also, participants with impairments in several IC domains plus cognition might have been exposed to a higher life course load of systemic inflammation than those without cognitive impairment. However, the interrelationships between IC domains in younger cohorts remains to be explored.

Moreover, the ‘opened gate’ for reversion in the high deterioration without cognition status was associated with lower mortality and dementia risk and better self-rated health than the low deterioration status. Still, it did not imply a lower risk of frailty or disability.

Our results are congruent with previous studies which applied the latent class model to IC [21, 22]. For example, Yu *et al.* [21] who also identified a group of healthy participants and two strata of deterioration. Interestingly, in their study, the nutrition, psychological and sensory domains also ‘move in block’. Our findings are consistent with those of Meng *et al.*, who found one class with no/few IC declines, another class with impairments in most IC domains and intermediate classes with impairments clustered around locomotion or cognition. Our study contributes new knowledge by extending the population heterogeneity and adding a longitudinal approach to describe transitions between states.

Our study has strengths. For instance, it includes older adults with harmonised measurements from low-and-middle-income regions across seven countries. Also, it is the first to apply a latent transition approach to IC, shedding light on its natural history within a 4–5-year period. Additionally, their association with a diversity of significant outcomes validated the clinical relevance of the latent statuses. Our study has limitations. The operationalisation of the IC impairments had to be adapted according to data availability and are slightly different from the ones recommended in the World Health Organization (WHO) ICOPE handbook. However, our IC measurement in the 10/66 study was recently validated [17]. Another limitation is the modification of Fried’s frailty phenotype (grip strength was unavailable). Yet, this modified frailty measurement has been tested with other frailty measures as a valid predictor of mortality, and dependence, amongst other outcomes [31, 33, 50].

The latent statuses we found are a closer representation of reality than previous studies of IC. The clinical team often meets people with more than one IC impairment in everyday life. We suggest that these IC clusters may help to design integrative management plans for older people. The current ICOPE guidelines treat each IC domain impairment separately and lack a strategy to integrate them. We suggest that our clusters could shed light on the matter of working simultaneously with more than one IC domain. Indeed, further research is needed amongst other populations.

The ‘low deterioration’ and ‘high deterioration without cognitive impairment’ groups seem ideal targets for intervention at the primary care level. For the low deterioration status (mainly due to mobility impairment), the goal would be to prevent the incidence of the other domain impairments. Notice that 27% of the older adults with this status might decline abruptly to the high deterioration with cognitive impairment status. The factors associated with this severe decline are unclear, but it could be due to nutritional, psychological or cognitive problems in the weakest physiological system. These results highlight the importance of intervening in older adults with impaired mobility, even if they do not manifest another IC domain impairment on the spot.

Interventions for the high deterioration without cognitive impairment status would need to be ideally aimed at reversing to the low deterioration status (e.g. 12% of the study population reversed). Or at least to avoid cognitive impairment because adding this condition might significantly worsen people’s prognosis.

In summary, our results provide a novel perspective of the natural history of IC declines with two levels of deterioration and strong binding of the nutritional and psychological domains, which deserves further research.

## Supplementary Material

aa-22-2165-File002_afad137

## Data Availability

The data underlying this study are restricted, as participants did not consent to sharing their information publicly. Data underlying the results presented in the study are available from the 10/66 Dementia Research Group public data archive for researchers who meet the criteria for access to confidential data. Information on procedures to request access is available at https://www.alz.co.uk/1066/1066_public_archive_baseline.php, or by contacting dementiaresearchgroup1066@kcl.ac.uk
