# Supplementary material for: Exploring the natural history of intrinsic capacity impairments: longitudinal patterns in the 10/66 study
Source: Age Ageing. 2023 Jul 29;52(7):afad137. doi: 10.1093/ageing/afad137 (PMC10387229; doi:10.1093/ageing/afad137)
Supplement: aa-22-2165-File002_afad137 [file aa-22-2165-file002_afad137.docx]

Appendix

Contents

[Supplementary material S1. Kaplan Meier survival estimates for the four latent statuses. 1](#_Toc133047897)

[Supplementary material S2. World Health Organization’s WHODAS 2.0 12-item instrument 2](#_Toc133047898)

[Supplementary material S3. Statistical details on the latent transitions model. 3](#_Toc133047899)

[Supplementary material S4. Marginal probabilities from the generalised ordered logistic model with self-rated health as the outcome. 10](#_Toc133047900)

[References 10](#_Toc133047901)

# Supplementary material S1. Kaplan Meier survival estimates for the four latent statuses.


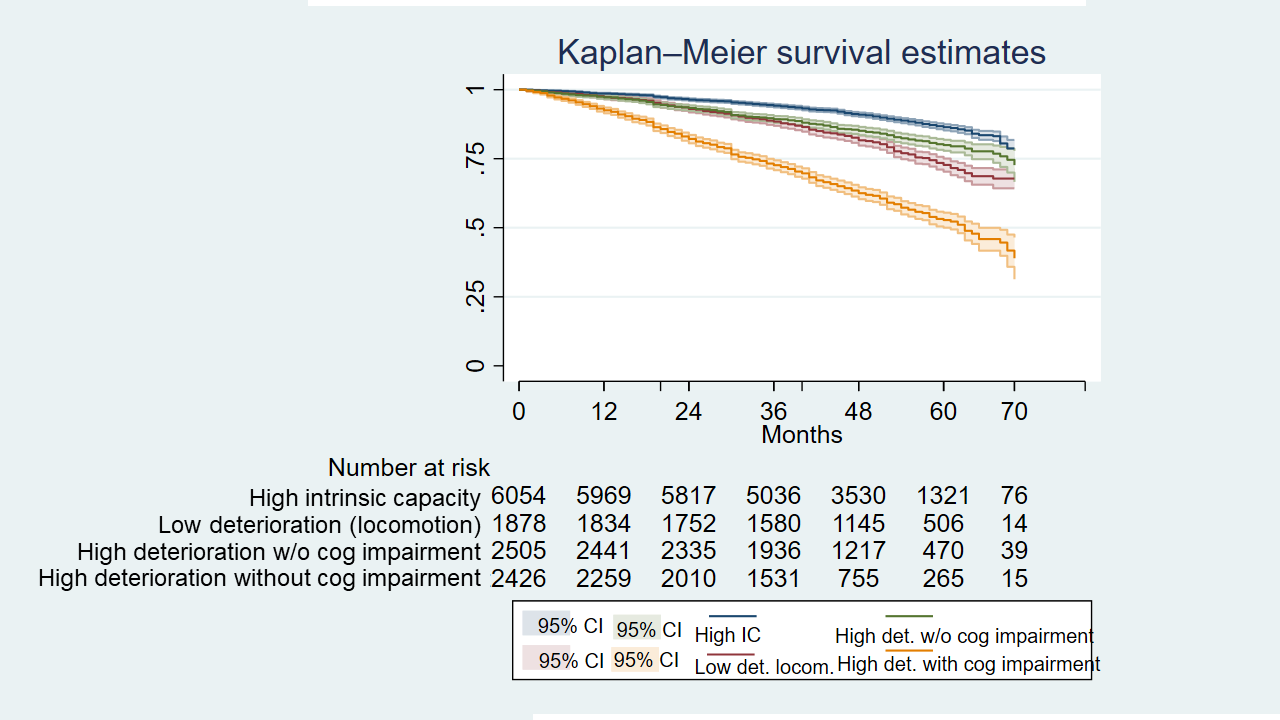


# Supplementary material S2. World Health Organization’s WHODAS 2.0 12-item instrument


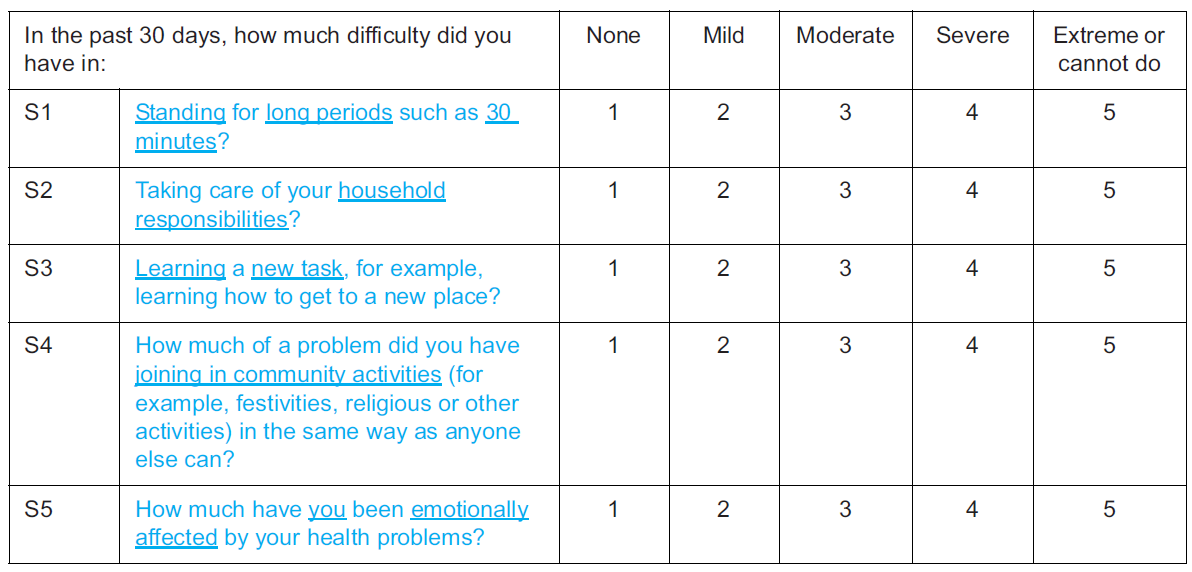

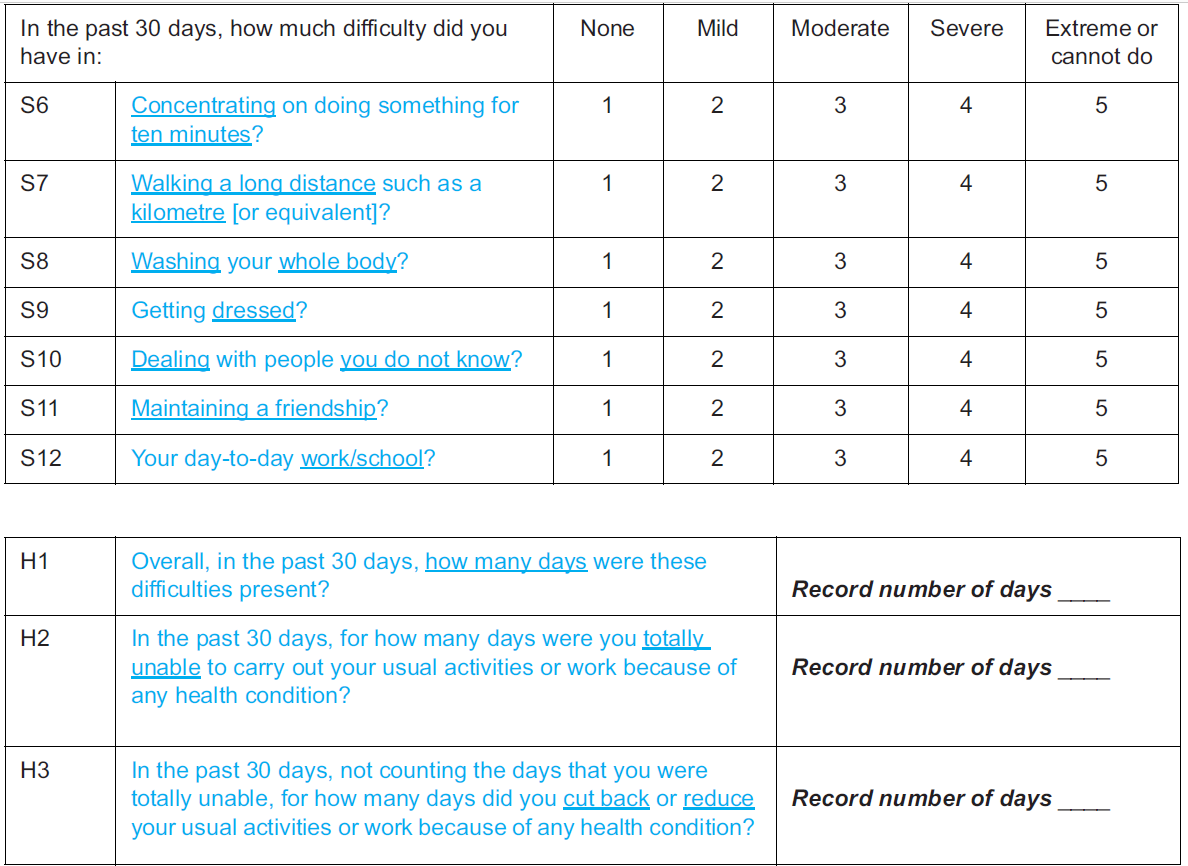


Extracted from: Measuring Health and Disability: Manual for WHO Disability Assessment Schedule (WHODAS 2.0) / edited by TB Üstün, N Kostanjsek, S Chatterji, J Rehm.^1^

# Supplementary material S3. Statistical details on the latent transitions model.

The purposes of applying the latent transition approach as a modality of finite mixture modelling to these domain impairments were to a) examine if intrinsic capacity impairments tend to present in clusters and b) analyse how older people transit from their baseline cluster at follow-up. Following conventional guidelines,^2^ we modelled it separately at each time point to explore the composition and the optimal number of classes cross-sectionally. We obtained Bayesian and Akaike information criterion (BIC and AIC) as the goodness of fit statistics for models with two, three, and four latent classes.

Third, we modelled the latent transitions using baseline and follow-up data simultaneously, going from two to five statuses to verify the goodness of fit. We restricted the model to have the identical composition of statutes at baseline and at follow-up for the transitions to be comparable. The observed indicator variables were the six dichotomous intrinsic capacity domain impairments, and we included age, sex, and education as covariates for the model. The estimated parameters were the prevalence of each of the statuses of the latent variable at baseline and follow-up (Table 2A); the probability of transitioning to the statuses conditional on belonging to each baseline status (Table 2B); the probability of belonging to each status conditional on exhibiting each intrinsic capacity domain impairment (Table 2C and Figure 1). We opted for the model with four statuses based on the physio-clinical correlation and the model diagnostics. We verified the latent status separation and homogeneity with model diagnostics: classification matrices, the goodness of fit statistics AIC and BIC, and the odds of correct classification^3^.

*Latent status model properties*

The model showed good latent class separation as expressed by the diagonal of posterior probabilities in the classification matrix with values >0.7 for all except for the low deterioration status (0.6).^4^ (Supplement S3.3) Also, the odds of correct classification were higher than 5.0 for all four statuses at baseline.^4^ The latent transition model with four statuses had an AIC=5574 and BIC=5871, compared to AIC=6546 and BIC=6744 for three statuses. (Supplementary material S3.2).

**S3.1 Cross-sectional latent statuses**

**
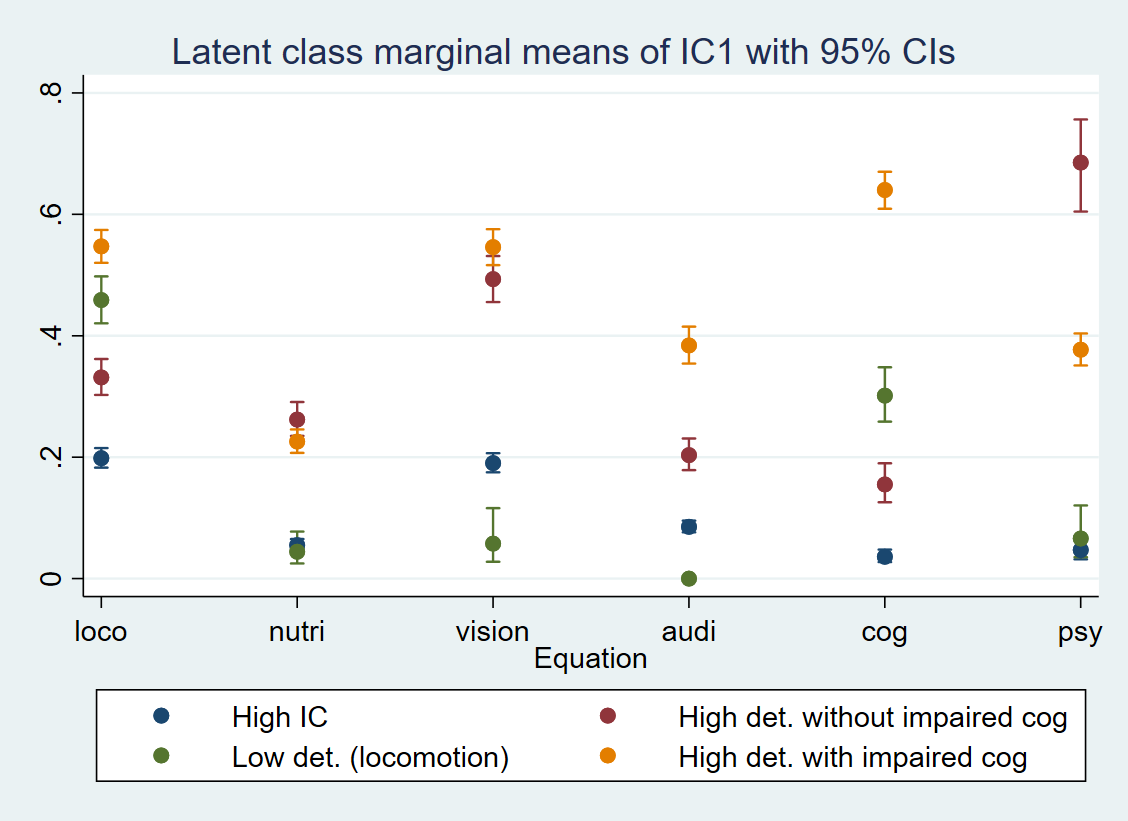
**

| **Baseline** | |  | **Follow-up** | |
| --- | --- | --- | --- | --- |
| **Class** | **Prevalence** |  | **Class** | **Prevalence** |
| **1** | **0.46** |  | **1** | **0.09** |
| **2** | **0.19** |  | **2** | **0.2** |
| **3** | **0.14** |  | **3** | **0.59** |
| **4** | **0.21** |  | **4** | **0.12** |

**S3.2 Goodness of fit statistics by number of latent statuses in the longitudinal models.**

| Number of latent statuses | 2 | 3 | 4 | 5 |
| --- | --- | --- | --- | --- |
| AIC | 103427 | 103017 | 5575 | 4838 |
| BIC | 103528 | 103172 | 5872 | 5249 |

**S3.3 Classification matrix for the latent transition models**

| Classification matrix | | | | |
| --- | --- | --- | --- | --- |
|  | High deterioration with cognitive impairment | Low deterioration mainly locomotion impairment | High deterioration without cognitive impairment | High intrinsic capacity |
| Baseline |  |  |  |  |
| High deterioration with cognitive impairment | 0.82 | 0.09 | 0.08 | 0.02 |
| Low deterioration mainly locomotion impairment | 0.08 | 0.63 | 0.10 | 0.19 |
| High deterioration without cognitive impairment | 0.07 | 0.05 | 0.74 | 0.13 |
| High intrinsic capacity | 0.01 | 0.11 | 0.09 | 0.80 |
| Total | 0.18 | 0.17 | 0.21 | 0.43 |
| Follow-up |  |  |  |  |
| High deterioration with cognitive impairment | 0.82 | 0.10 | 0.08 | 0.01 |
| Low deterioration mainly locomotion impairment | 0.07 | 0.65 | 0.10 | 0.18 |
| High deterioration without cognitive impairment | 0.10 | 0.14 | 0.69 | 0.06 |
| High intrinsic capacity | 0.00 | 0.41 | 0.06 | 0.53 |
| Total | 0.26 | 0.41 | 0.19 | 0.15 |
|  |  |  |  |  |
| Odds of correct classification | | | | |
| Baseline | 19.9 | 8.5 | 10.5 | 5.2 |
| Follow-up | 13.1 | 2.8 | 9.9 | 6.3 |

Classification matrix: displays the mean probability of being in the baseline (row) status given your belonging to the follow-up (column) status. (Recommended >0.7) ^4^

Odds of correct classification: compared the odds of being correctly classified given the posterior probability of membership to the odds of randomly being classified to that status. (Recommended >5.0) ^4^

**S3.4 Latent transitions model using frailty as a covariate (suggested by Reviewers)**

*Table S3.4.1 Prevalence of latent statuses*

| Baseline | |  | Follow-up | |
| --- | --- | --- | --- | --- |
| Class | Prevalence |  | Class | Prevalence |
| High intrinsic capacity | 0.48 |  | High intrinsic capacity | 0.29 |
| Low deterioration (locomotion) | 0.19 |  | Low deterioration (locomotion) | 0.16 |
| High deterioration without cognitive impairment | 0.14 |  | High deterioration without cognitive impairment | 0.28 |
| High deterioration with cognitive impairment | 0.19 |  | High deterioration with cognitive impairment | 0.27 |

*Fig S3.4 Intrinsic capacity domains affected per latent status*

*Table S3.4.2 Transition Matrix*

|  | high deterioration with cognitive impairment | High deterioration without cognitive impairment | Low deterioration mainly locomotion impairment | High IC |
| --- | --- | --- | --- | --- |
| high deterioration with cognitive impairment | 1 | 0 | 0 | 0 |
| High deterioration without cognitive impairment | 0.09 | 0.61 | 0.00 | 0.30 |
| Low deterioration mainly locomotion impairment | 0.09 | 0.19 | 0.58 | 0.14 |
| High IC | 0.11 | 0.33 | 0.10 | 0.46 |

Goodness of fit: with frailty Log-likelihood: -67343.76;
without frailty, Log-likelihood: -70528.23

**S3.5 Cox models for mortality stratified by country (as suggested by reviewers)**

|  | aHR | p | 95%CI | |
| --- | --- | --- | --- | --- |
| Cuba | n=2,285 |  |  |  |
| Low deterioration (locomotion) | 0.99 | 0.928 | 0.71 | 1.37 |
| High deterioration w/o cog impairment | 1.45 | 0.005 | 1.12 | 1.89 |
| High deterioration with cog impairment | 1.81 | <0.001 | 1.34 | 2.43 |
| Dominican Republic | n=1,510 |  |  |  |
| Low deterioration (locomotion) | 1.51 | 0.024 | 1.06 | 2.16 |
| High deterioration w/o cog impairment | 1.06 | 0.723 | 0.75 | 1.50 |
| High deterioration with cog impairment | 1.78 | 0.003 | 1.21 | 2.62 |
| Peru | n=1,730 |  |  |  |
| Low deterioration (locomotion) | 1.36 | 0.388 | 0.68 | 2.71 |
| High deterioration w/o cog impairment | 1.38 | 0.217 | 0.83 | 2.32 |
| High deterioration with cog impairment | 2.34 | 0.008 | 1.24 | 4.40 |
| Venezuela | n=1,641 |  |  |  |
| Low deterioration (locomotion) | 1.55 | 0.160 | 0.84 | 2.84 |
| High deterioration w/o cog impairment | 1.49 | 0.073 | 0.96 | 2.30 |
| High deterioration with cog impairment | 3.09 | <0.001 | 1.86 | 5.16 |
| Mexico | n=1,841 |  |  |  |
| Low deterioration (locomotion) | 1.19 | 0.520 | 0.70 | 2.01 |
| High deterioration w/o cog impairment | 1.13 | 0.598 | 0.72 | 1.77 |
| High deterioration with cog impairment | 1.35 | 0.233 | 0.83 | 2.20 |
| China | n=1,791 |  |  |  |
| Low deterioration (locomotion) | 1.22 | 0.161 | 0.92 | 1.60 |
| High deterioration w/o cog impairment | 1.38 | 0.136 | 0.90 | 2.10 |
| High deterioration with cog impairment | 1.84 | 0.001 | 1.29 | 2.63 |
| Puerto Rico | n=1,443 |  |  |  |
| Low deterioration (locomotion) | 1.46 | 0.092 | 0.94 | 2.27 |
| High deterioration w/o cog impairment | 0.89 | 0.563 | 0.59 | 1.33 |
| High deterioration with cog impairment | 1.82 | 0.003 | 1.23 | 2.68 |

aHR= hazard ratio adjusted for age, sex, education, and comorbidities.

# Supplementary material S4. Marginal probabilities from the generalised ordered logistic model with self-rated health as the outcome.

| Adjusted* marginal probabilities of rating health as very good | | | | | | | |  |
| --- | --- | --- | --- | --- | --- | --- | --- | --- |
| n= 14,904 | Pr | | 95%CI | | | | |  |
| High intrinsic capacity | | 0.184 | | (0.174 | | ; | 0.194) | |
| Low deterioration mainly locomotion impairment | | 0.136 | | (0.131 | | ; | 0.142) | |
| High deterioration without cognitive impairment | | 0.099 | | (0.092 | | ; | 0.106) | |
| High deterioration with cognitive impairment | | 0.072 | | (0.063 | | ; | 0.080) | |
|  |  | |  | |  | |  |  |
| Adjusted* marginal probabilities of rating health as very bad | | | | | | | |  |
| n= 14,904 | Pr | | 95%CI | | | | |  |
| High intrinsic capacity | | 0.003 | | (0.002 | | ; | 0.003) | |
| Low deterioration mainly locomotion impairment | | 0.007 | | (0.005 | | ; | 0.009) | |
| High deterioration without cognitive impairment | | 0.019 | | (0.016 | | ; | 0.022) | |
| High deterioration with cognitive impairment | | 0.050 | | (0.040 | | ; | 0.061) | |

*Adjusted for age, sex and education

# References

1. Üstün, T. B. *et al.* Developing the world health organization disability assessment schedule 2.0. *Bull. World Health Organ.* **88**, 815–823 (2010).

2. Collins, L. M. & Lanza, S. T. *Latent Class and Latent Transition Analysis: With Applications in the Social, Behavioral, and Health Sciences*. (John Wiley & Sons, Inc, 2009). doi:10.1002/9780470567333.

3. Nagin, D. *Group-Based Modeling of Development*. (2005).

4. Nagin, D. S. & Odgers, C. L. Group-based trajectory modeling in clinical research. *Annu. Rev. Clin. Psychol.* **6**, 109–138 (2010).
